# Phantom limb pain: thinking outside the (mirror) box

**DOI:** 10.1093/brain/awab139

**Published:** 2021-03-31

**Authors:** Tamar R Makin

**Affiliations:** Institute of Cognitive Neuroscience, University College London, London, UK

## Abstract

Despite our best efforts over the past century, our mechanistic understanding of phantom limb pain and our ability to treat it have remained limited. Tamar Makin invites readers to think more critically about some of the most popular approaches to understanding and treating this condition.

***It has long been established that phantom limb pain is a real physiological condition. Why then do we tolerate mystery and myth when it comes to phantom limb pain treatment?***

Following amputation, individuals generally report experiencing vivid sensations of their missing limb, which for the majority of people may also feel painful. Phantom limb pain (PLP) is a curious phenomenon; we find it interesting because it raises challenging questions relevant to what it means for us to live inside our bodies, and has thus been a source of wonder and curiosity throughout modern culture. René Descartes liked to use PLP as a cautionary example for why the human senses cannot be trusted. Admiral Nelson, who lost his arm in 1797, took his phantom sensations as evidence for the existence of his eternal soul. More recently, millions of viewers sympathized with the struggles of patients with PLP and their medical teams on hit TV shows, such as *Grey’s Anatomy* and *House*, and PLP has even been the topic of an action-adventure stealth video game. Alongside popular culture, PLP has also inspired a plethora of clinical speculation and research. 

PLP was first clinically characterized in 1551 by one of the forefathers of modern surgery, Ambroise Paré:

*‘Verily it is a thing wondrous strange and prodigious, and which will scarce be credited, unless by such as have seen with their eyes, and heard with their ears the Patients who have many months after the cutting away of the Leg, grievously complained that they yet felt exceeding great pain of that leg so cut off’.*

Silas Weir Mitchell, a US Civil War surgeon, coined the term ‘phantom pain’, which he described as: ‘*these hallucinations … so vivid so strange*’.

But for a person suffering from PLP, these sensations are tangible. One amputee, who lost her arm to cancer, describes her sensations:

‘*To anyone looking at me, I have no arm. But I can feel the entirety of my phantom hand and arm. Imagine you are wearing an elbow length evening glove … everywhere the glove touches your skin it’s crushing your arm constantly. … On top of it you get pains like burning pains. It’s like when you burn yourself on the grill. Your instinct is to pull your hand away, but with this pain you can’t. It's a nerve sensation and it stays there, until “it” decides to pull away*’.

Although originally considered to be rare,^1^ most recent accounts estimate the incidence of PLP among those who have undergone limb amputation at ∼63% [95% confidence interval (CI): 58.23–67.05].^2^ Despite being very common, PLP is notoriously difficult to treat with conventional medicine.^3^ The unusual challenge we are faced with is that the body part to be treated is not physically present. A mechanistic understanding of the neural basis of PLP is thus needed to treat it successfully.

Why do people experience PLP? Early observations showing that PLP can be evoked by applying pressure to the stump led to the theory that it may relate to the peripheral nerves. This was elegantly demonstrated using intraneural recordings from the residual limb of people who had undergone an amputation: even though the receptors of the peripheral nerve are missing, the residual axons still generate and transmit action potentials. Importantly, both spontaneous and evoked PLP are reflected in the electrical activity of the residual nerve.

This finding inspired a simple mechanistic explanation for PLP: as these peripheral nerves normally provide information about touch and pain originating from the hand, inputs provided by these nerves will be interpreted by the CNS as arising from the missing hand. Clinical attempts to use local anaesthesia to block this ectopic electrical activity proved difficult to implement,^4^ potentially due to the challenges associated with long-term blocking of nociceptive C-fibres. However, blocking any peripheral signals to the CNS by applying local anaesthesia to the cell body, produced rapid and reversible attenuation—and often complete elimination—of PLP. This provides a powerful demonstration that PLP originates in the periphery.

And yet this simple mechanism has been largely marginalized in comparison to more ambiguous explanations based on psychopathology or cortical neural mechanisms.

Many theories dominating the early 20th century assumed that PLP was neurotic in nature, manifested by ‘denial’ or even ‘hysteria’.^1^ For example, R.D. Langdale Kelham, a pioneer in post-amputation rehabilitation concluded that the typical patient with a phantom limb was, more often than not, someone with an ‘unsatisfactory personality’:

‘*It may be he is an anxious, introspective, dissatisfied, ineffective [sic] who, becoming obsessed by his symptoms, and brooding upon them and his disability, tends to dramatise their degree, using undoubted exaggerations in his description of his sufferings*’.

Theories relying on psychopathology or other psychogenic mechanisms to explain PLP have been conclusively debunked. Cognitive behavioural therapy, however, is a common tool for helping amputees cope with the consequences of PLP.^3^

Others have considered the anatomical origins of PLP to lie in the sensorimotor CNS. This possibility paved the way for a flurry of surgical interventions in the latter half of the 20th century, ranging from antero-lateral chordotomy to ablation of the postcentral gyrus, with relatively poor clinical outcomes (Fig. 1).

**Figure 1:**
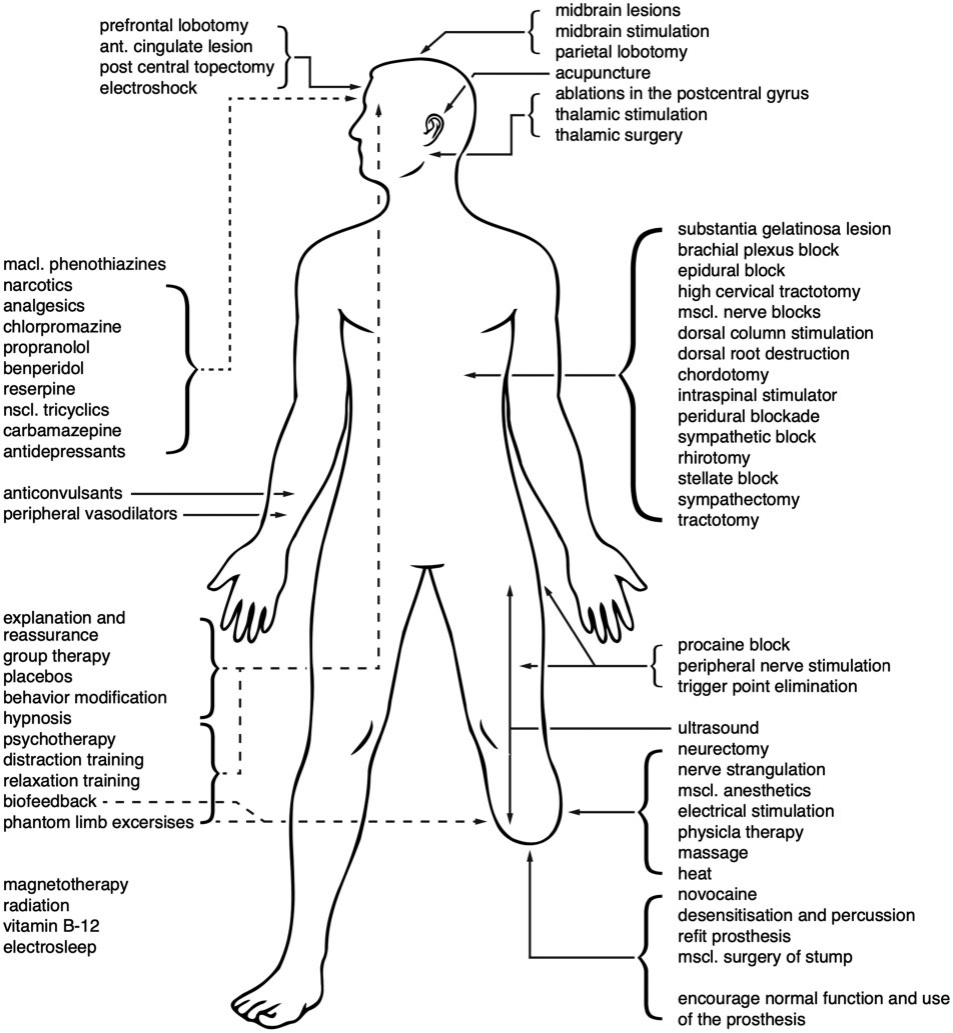
**PLP treatments.** Reproduced with permission from Sherman *et al*.^4^

Since the end of the 20th century, the prevailing theory for the development of PLP has been that of maladaptive brain plasticity. This idea is based on an observation in monkeys where loss of input to the brain’s hand area (e.g. following arm deafferentation) leads to redistribution of brain resources, termed brain plasticity, or reorganization. Simply put, the cortical resources of the (now missing) hand become freed up, and subsequently get taken over by a new body part. Intuitively, you might expect that the brain’s ability to reassign resources across body parts based on altered demand should be helpful, and perhaps even allow people who have lost a limb to better adapt to their disability. An example of adaptive plasticity would be early-blind individuals, where the visual cortex becomes involved in non-visual processing for perception and language.

However, according to the maladaptive plasticity theory of PLP, reorganization in the adult brain can be harmful. This idea is rooted in an observation that a relatively crude measure of brain reorganization in amputees correlates with PLP.^5^ Consequently, cortical reorganization was proposed to trigger pain in the phantom hand as a result of the cortical area corresponding to the missing limb becoming activated by the invading inputs. This input mismatch was thought to generate an ‘error’ signal that is interpreted by the brain as pain arising from the missing hand. This theory provides clear predictions on how to treat PLP: if pain is caused by maladaptive reorganization, then we need to reverse the reorganization to alleviate PLP.

Presently, some of the most widely used treatments for PLP aim to reverse maladaptive plasticity by ‘reinstating’ the representation of the missing hand to its original territory.^3^ Mirror box therapy uses illusory visual information about the missing hand (by reflecting an image of the intact hand via a mirror), in an effort to restore the missing hand representation in primary somatosensory cortex (Fig. 2). A related approach, using implicit and explicit motor imagery, aims to gradually ‘reawaken’ the motor representation of the missing hand. Building on these ideas, virtual reality approaches aim to improve phantom motor execution in an attempt to ‘normalize’ the sensorimotor representation of the missing hand. Common to these and other techniques is the ambition to exploit neuroplasticity mechanisms to reinstate normal sensory and motor representations.

**Figure 2:**
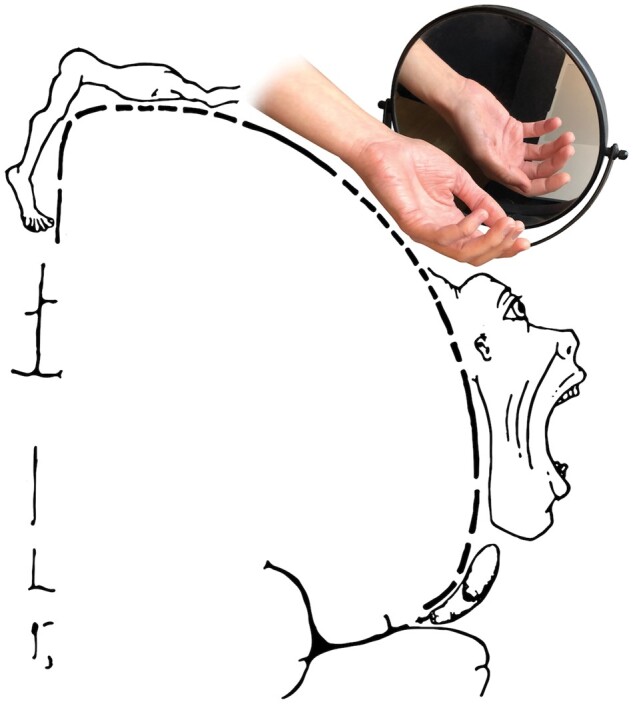
**Mirror box treatment aims to reinstate the representation of the missing hand in order to reverse maladaptive brain plasticity.** Modified from ‘Motor homunculus' by Ralf Stephan, ralf@ark.in-berlin.de, https://commons.wikimedia.org/wiki/File:Motor_homunculus.svg (22 March 2021, date last accessed) licensed under CC BY-SA 4.0, https://creativecommons.org/licenses/by-sa/4.0, via Wikimedia Commons.

Although the idea of a neuroscientific mechanism may be compelling, it is important to remember that we should not infer causation from a correlation, such as that observed between reorganization and PLP. A closer examination of the maladaptive theory and its ensuing therapies reveals a number of unsupported assumptions and a consistent lack of efficacy, respectively.

Consider some of the key hypotheses underlying the maladaptive theory. First, the notion that input loss ‘erases’ the representation of the missing hand, leading to cortical reorganization, has been negated.^6^ Recent human studies using advanced neuroimaging techniques fail to find invasion of foreign inputs into the cortical territory of the missing hand.^7^ Instead, multiple lines of evidence demonstrate that the brain retains the representation of the missing hand despite the fact that the hand is physically absent. In other words, there is no need to ‘reinstate’ the representation of the hand, which persists after amputation. As a side note, you cannot trick somatosensory cortex into reorganizing with visual information, simply because visual input is not a powerful modulator of this particular brain area.

Second, the idea that a foreign input causes pain by triggering an error signal in the cortex representing the missing hand has also long been refuted.^6^ For example, researchers have artificially stimulated the somatosensory hand territory in a deafferented patient, by injecting very small currents directly into the brain. According to the maladaptive theory, this should result in an ‘error’ signal, potentially giving rise to pain. But instead, this procedure triggers tactual and non-painful sensations on the insensate hand. Therefore, consistent with other results from studies using stimulation in amputees’ motor cortex, displaced inputs to the missing hand territory do not cause pain. Instead, pain sensations are better linked to a set of brain areas with a connectome distinct from that of the sensorimotor network.

Perhaps the most compelling evidence against the maladaptive plasticity theory is its poor clinical outcomes. As stated, over the past three decades, the maladaptive theory has become assertively dominant. For example, the original paper reporting a correlation between cortical plasticity and PLP has been cited almost 2000 times.^5^ Consequently, its therapeutic derivatives have dominated clinical practice—according to a recent international survey, four of the six most widely recommended PLP treatments (including both pharmacological and non-pharmacological options) are based on reversing maladaptive plasticity.^3^ Yet, PLP is still a common condition, and the overwhelming consensus across clinical trials, systematic reviews and meta-analyses is that there is no strong evidence that these clinical approaches provide consistent and long-lasting PLP relief, beyond a placebo control.^8^

Then why are we continuing to use these unsuccessful therapies? The limited efficacy of these therapies is exhasperated by the fact that much of the first-level evidence supporting these treatments is compromised. To begin with, PLP and its relief are ultimately measured by subjective report, which is fundamentally susceptible to suggestion and biases. Without a direct comparison to a double-blind placebo-controlled study arm, any observed changes in PLP reports should be treated with scepticism. Yet, this gold standard is rarely adopted in PLP research.

A further challenge is that PLP phenomenology is diverse, and therefore studies aimed at tracking PLP tend to use multiple pain scales. This becomes a problem when researchers ‘cherry-pick’ a particular outcome measure *post hoc*, without accounting for the multiple potential comparisons that have been performed.

A third problem relates to the mechanisms of pain alleviation. Some of the newest virtual/augmented reality treatments use principles from gamification to make the therapy more engaging, but attentional distraction is known to have pain-relieving benefits.^9^ Let’s consider an ongoing clinical trial, where phantom movements are used to control a video game in a virtual/augmented environment.^10^ This intervention is compared to a control condition where participants are asked to imagine moving the phantom but not engage in or control the game. Any benefits incurred by the main treatment might be attributable to distraction arising from this increased engagement.

Considering that no effective PLP treatment is currently available, one might argue that there is no harm in providing patients with suboptimal treatments. Indeed, the placebo effect is extremely powerful, and could be harnessed to ease the suffering of individuals struggling with intractable pain. But we should also consider the consequences of deliberately developing and using suboptimal treatments. From an ethical standpoint, if we know the treatment is not more effective than a placebo, we should make this explicitly clear to the patient and the clinical team. This is especially true when the treatment might be expensive or time consuming for the patient.

From a policy perspective, the development and assessment of mirror box-like treatments has consumed an enormous share of the resources available to the small community tasked with developing targeted PLP treatments. This leaves very few research and innovation opportunities for identifying alternative and potentially more successful treatments, which we desperately need. Rather than rehashing unsuccessful treatments, we should instead work towards practical methods to suppress the PLP generators that have been identified.

In their very detailed and comprehensive account of phantom limbs from 1948, Henderson and Smyth concluded:

‘*To put the matter briefly, that which still exists is working in harmony with that which has ceased to exist* ***except as a pattern in the cortex***’ [emphasis in original text].

It appears that despite our best efforts over the past 70 years, our mechanistic conceptualization of PLP and its treatment have not advanced much beyond this vague notion. Unfortunately, at this point, we still don’t have a consensus understanding of the neural drivers of PLP. We don’t even know if this condition is mechanistically any different from other more common, and arguably less romanticized, chronic pain conditions, such as joint pain. But considering how futile our focus on maladaptive brain plasticity has been so far, it is time for us to shed our romantic prenotions around this pain condition, and start thinking outside the (mirror) box.

## Notes

Tamar Makin is a Professor of Cognitive Neuroscience at UCL and leader of the London Plasticity Lab https://plasticity-lab.com/
